# Cyclin dependent kinase inhibitor 3 (*CDKN3*) upregulation is associated with unfavorable prognosis in clear cell renal cell carcinoma and shapes tumor immune microenvironment: A bioinformatics analysis

**DOI:** 10.1097/MD.0000000000035004

**Published:** 2023-09-08

**Authors:** Ahmed H. Al Sharie, Abdulmalek M. Abu Zahra, Tamam El-Elimat, Reem F. Darweesh, Ayah K. Al-Khaldi, Balqis M. Abu Mousa, Mohammad S. Bani Amer, Yazan O. Al Zu’bi, Kinda Al-Kammash, Alma Abu Lil, Abubaker A. Al Malkawi, Zainab Alazzeh, Feras Q. Alali

**Affiliations:** a Faculty of Medicine, Jordan University of Science & Technology, Irbid, Jordan; b Department of Medical Laboratory Sciences, Jordan University of Science and Technology, Irbid, Jordan; c Department of Medicinal Chemistry and Pharmacognosy, Faculty of Pharmacy, Jordan University of Science and Technology, Irbid, Jordan; d College of Pharmacy, QU Health, Qatar University, Doha, Qatar.

**Keywords:** bioinformatics, ccRCC, CDKN3, clear cell renal cell carcinoma, cyclin-dependent kinase inhibitor 3, TCGA

## Abstract

Cell cycle regulatory proteins plays a pivotal role in the development and progression of many human malignancies. Identification of their biological functions as well as their prognostic utility presents an active field of research. As a continuation of the ongoing efforts to elucidate the molecular characteristics of clear cell renal cell carcinoma (ccRCC); we present a comprehensive bioinformatics study targeting the prognostic and mechanistic role of cyclin-dependent kinase inhibitor 3 (*CDKN3*) in ccRCC. The ccRCC cohort from the Cancer Genome Atlas Program was accessed through the UCSC Xena browser to obtain *CDKN3* mRNA expression data and their corresponding clinicopathological variables. The independent prognostic signature of *CDKN3* was evaluated using univariate and multivariate Cox logistic regression analysis. Gene set enrichment analysis and co-expression gene functional annotations were used to discern *CDKN3*-related altered molecular pathways. The tumor immune microenvironment was evaluated using TIMER 2.0 and gene expression profiling interactive analysis. *CDKN3* upregulation is associated with shortened overall survival (hazard ratio [HR] = 2.325, 95% confident interval [CI]: 1.703–3.173, *P* < .0001) in the Cancer Genome Atlas Program ccRCC cohort. Univariate (HR: 0.426, 95% CI: 0.316–0.576, *P* < .001) and multivariate (HR: 0.560, 95% CI: 0.409–0.766, *P* < .001) Cox logistic regression analyses indicate that *CDKN3* is an independent prognostic variable of the overall survival. High *CDKN3* expression is associated with enrichment within the following pathways including allograph rejection, epithelial–mesenchymal transition, mitotic spindle, inflammatory response, IL-6/JAK/STAT3 signaling, spermatogenesis, TNF-α signaling via NF-kB pathway, complement activation, KRAS signaling, and INF-γ signaling. *CDKN3* is also associated with significant infiltration of a wide spectrum of immune cells and correlates remarkably with immune-related genes. *CDKN3* is a poor prognostic biomarker in ccRCC that alters many molecular pathways and impacts the tumor immune microenvironment.

## 1. Introduction

Primary malignant renal tumors represent an aggressive and heterogenous group of malignancies. The most prevalent type is renal cell carcinoma (RCC), which constitutes around 1% to 3% of all cancers and 90% of kidney neoplasms.^[1,2]^ It is the seventh most common cancer in males and the ninth most common in females. Globally, there is a 3:2 male predominance, with a peak incidence in the sixties and seventies.^[3]^ The prognosis of RCC patients varies considerably based on age, clinical stage, and pathological classification.^[4]^ During the past decade, a pressing need to explore new biomarkers and potential therapeutic targets has emerged. RCC risk factors include obesity, hypertension, and smoking.^[5]^ These factors are widely recognized as major contributors to RCC. Although some studies have suggested other associations with RCC, including a possible protective effect of alcohol consumption, a negative effect of red meat consumption, and occupational exposure to carcinogens, the data on these factors and their relationship to RCC are not fully elucidated.^[6–8]^

Approximately 97% of RCC cases occur sporadically, devoid of any family history or genetic predisposition, while the remaining 2% to 3% have been identified due to causative genetic events.^[9]^ Generally, structural alterations of the short arm of chromosome 3 are observed in RCC patients.^[10]^ To date, certain genome-wide association studies of RCC have identified 6 susceptibility loci on chromosome regions 2p21, 2q22.3, 8q24.21, 11q13.3, 12p11.23, and 12q24.31.^[11–14]^ Moreover, genetic studies on familial RCC have revealed some critical genetic mutations in a number of genes involved in different cellular processes (*SETD2, KDM5C, PBRM1, BAP1, FLCN, FH, MET, PTEN, SDHB, SDHC, SDHD, TSC1, TSC2*, and *VHL*), which have also been linked to the development of some sporadic RCC cases.^[15,16]^ Some of these genetic variations have been associated with distinct autosomal dominant syndromes each with its own genetic basis and phenotypic manifestations. Among them, von Hippel-Lindau syndrome, hereditary papillary renal cell carcinoma, Birt-Hogg-Dubé syndrome, hereditary leiomyomatosis cell carcinoma, and tuberous sclerosis.

Aberrant expression of cell cycle regulatory genes plays a vital role in cancer progression. These regulatory elements are considered potential survival markers and therapeutic targets. There is increasing evidence that cyclin-dependent kinase inhibitor 3 (CDKN3) has significant regulatory roles in cancer development. *CDKN3* gene is mapped to14q22 encoding the CDKN3 protein. It belongs to the dual-specificity protein phosphatase family, which possesses dephosphorylation activity by interacting with CDK2 kinase at Thr160, thus preventing its activation. Due to the role of Thr160 phosphorylation in activating CDK2 and promoting cell cycle progression, CDKN3 upregulation leads to the suppression of G1-S phase transition. Further, it is recognized as an MDM2 binding protein that can form a complex with p53 and MDM2, leading to the repression of p21 induction and the promotion of the cell cycle. Conversely, Srinivas et al^[17]^ revealed that cell cycle inhibition is controlled by binding to the MSP1 region of the centrosome and blocking the formation of abnormal spindles. Previous studies have reported different expression patterns of CDKN3 in several types of cancers. However, the effect of CDKN3 on tumorigenesis and the molecular mechanisms involved remains unclear. In the majority of cancer types, CDKN3 functions as an oncogene and its gene expression is upregulated, including, nasopharyngeal,^[18]^ cervical,^[19]^ lung,^[20]^ prostate,^[21]^ breast,^[22]^ esophageal,^[23]^ liver,^[24]^ and renal cancer cells.^[25]^ Thus, it promotes tumorigenesis and is negatively correlated with survival. However, studies on gastric cancer, leukemic, and glioblastoma cell lines found that *CDKN3* overexpression has been associated with the inhibition of cell proliferation and metastasis promotion.^[26–28]^ In contrast, CDKN3 is a tumor suppressor gene and found to be downregulated in brain tumors.^[29]^

The use of bioinformatics in oncology has become a prevalent multidisciplinary tool to uncover potential genetic targets implicated in the development of neoplasm and to assess drug responsiveness. Such an approach can provide valuable insights into the molecular mechanisms of tumor pathogenesis, central signaling pathways, and cellular activity networks involved in RCC. In the current study, a comprehensive bioinformatics analysis evaluating the prognostic potential of *CDKN3* expression in RCC has been conducted.

## 2. Materials and methods

### 2.1. Clinical and transcriptomic data acquisition and processing

The main objective of this work is to investigate the prognostic potential of *CDKN3* mRNA expression in ccRCC patients. Cancer Genome Atlas Program (TCGA) level 3 expression data along with the corresponding clinicopathological variables were retrieved using the California Santa Cruz Cancer Genomics Browser (UCSC Xena, http://xena.ucsc.edu/).^[30]^ *CDKN3* expression from tumor samples and normal tissues adjacent to the tumor (NAT) were obtained in a normalized RNA-Seq by expectation maximization count transformed as log_2_(x + 1). Clinicopathological parameters include age, gender, International Society of Urologic Pathologists grade, TNM scoring system, and the American Joint Committee on Cancer stage. The primary endpoints were the overall survival (OS) and progression-free interval (PFI). The ccRCC cohort was manually curated by omitting patients with OS time of 0 and patients without *CDKN3* expression data. ccRCC cohort was also divided into 2 subsets based on *CDKN3* expression (high vs how expression) with a cut-point determined by X-tile software as previously described.^[31]^

### 2.2. Statistical analysis

Statistical analysis was performed as previously described.^[32]^ In brief, IBM SPSS statistical package for Windows v.26 (Armonk, NY) and GraphPad Prism v.9.3.1 (San Diego, CA) were utilized for conducting statistical analyses and generating graphs. The presentation of nominal data included frequency (percentage). Normally distributed continuous variables were represented using mean ± standard deviation of the mean or standard error of the mean, while non-normally distributed data were described using median (interquartile range). The assessment of normality involved the Kolmogorov-Smirnov test, Shapiro–Wilk test, and analysis of quantile-quantile plots. To analyze the relationship between *CDKN3* expression status and clinicopathological variables, several statistical tests were utilized. Categorical variables were evaluated using the *Chi*-square test or Fisher exact test. Paired *t*-test was applied for normally distributed paired samples, and unpaired *t*-test and Welch corrected unpaired *t*-test were used for normally distributed non-paired samples, taking into account variance equality. Non-normally distributed data were analyzed using Wilcoxon matched pairs test and Mann–Whitney *U*-test.

Kaplan–Meier survival methods were employed to examine the impact of *CDKN3* expression status on OS and PFI. The log-rank test was used to identify statistical differences across survival curves, and the outcomes were reported with 95% confidence intervals (95% CI) and hazard ratios (HR). *CDKN3* expression data were evaluated for predicting the aforementioned endpoints. The predictability power was evaluated with receiver operating curve analysis. Univariate and multivariate Cox logistic regression models were applied to assess the independent prognostic significance of clinicopathological variables and *CDKN3* expression. Prior to Cox logistic regression analysis, the variables were dichotomized as follows: age (reference: ≤53 years), gender (reference: female), pT stage (reference: T1 + T2), pN stage (reference: N0), American Joint Committee on Cancer stage (reference: stage 1 + 2), ISUP grade (reference: grade 1 + 2), and *CDKN3* expression (reference: low). All statistical tests conducted were two-sided, and a significance level of *P* ≤ .05 was considered to indicate statistical significance.

### 2.3. Gene set enrichment analysis (GSEA)

To investigate the underlying molecular mechanisms related to the prognostic *CDKN3* signature, a specialized GSEA was conducted.^[33,34]^ GSEA involved a comprehensive exploration of gene expression patterns, comparing 2 distinct groups based on their *CDKN3* expression levels (high vs low) using the C1 gene sets (hallmarks of cancer). The GSEA analysis incorporated 1000 permutations using the “gene set” permutation type, utilizing the Human_UniProt_IDs chip platform. Significance of gene set enrichment was determined by an adjusted *P* value of ≤ .001 and a false discovery rate (FDR) below 0.25.

### 2.4. Protein–protein interaction (PPI) network construction

Retrieval and construction of *CDKN3*-related interacting genes were browsed using STRING v.11 (https://string-db.org).^[35]^ Significant interactions were labeled if a combined score ≥ 0.4 was observed based on phylogenetic co-occurrence, homology, co-expression, experimentally determined interaction, and automated text mining scoring. The maximum number of interactions was limited to 50.

### 2.5. LinkedOmics database analysis

The LinkedOmics database (http://www.linkedomics.org/login.php) was accessed to analyze *CDKN3*-related co-expressed genes within the TCGA ccRCC cohort.^[36]^ A gene was considered statistically significant with a Spearman correlation’s *P*-value ≤ .05 and FDR ≤ .05. Co-expressed genes were functionally annotated using GSEA within the gene ontology (GO) terms including biological process, molecular function, cellular component. In addition, the Kyoto encyclopedia of genes and genomes (KEGG) pathway classification and micro-RNA (micro-ribonucleic acid) target prediction ports were applied as well. The rank criteria were set to the *P*-value with a minimal number of 3 genes per set and 500 simulations.

### 2.6. Immune infiltration analysis

Tumor immune infiltration was evaluated using TIMER 2.0 (https://cistrome.shinyapps.io/timer/); a comprehensive resource that provides a systematic and extensive analysis of immune infiltrates across a wide spectrum of human malignancies.^[37]^ TIMER functions through a well-established statistical methodology known as deconvolution to infer the abundance of tumor-infiltrating immune cells from gene expression profiles. Immune components infiltration was reported using Spearman correlation coefficient and its associated *P*-value.

### 2.7. Immune genes correlation analysis using gene expression profiling interactive analysis

Selected genes known to influence significantly correlated immune cells were subjected to correlation analysis with *CDKN3* expression using gene expression profiling interactive analysis (http://gepia.cancer-pku.cn/index.html).^[38]^ The correlation was reported with Spearman correlation coefficient and its associated *P*-value within the TCGA ccRCC, TCGA NAT, and GTEx normal kidney cortex cohorts.

## 3. Results

### 3.1. *CDKN3* is highly expressed across a panel of human malignancies

**Figure 1.**
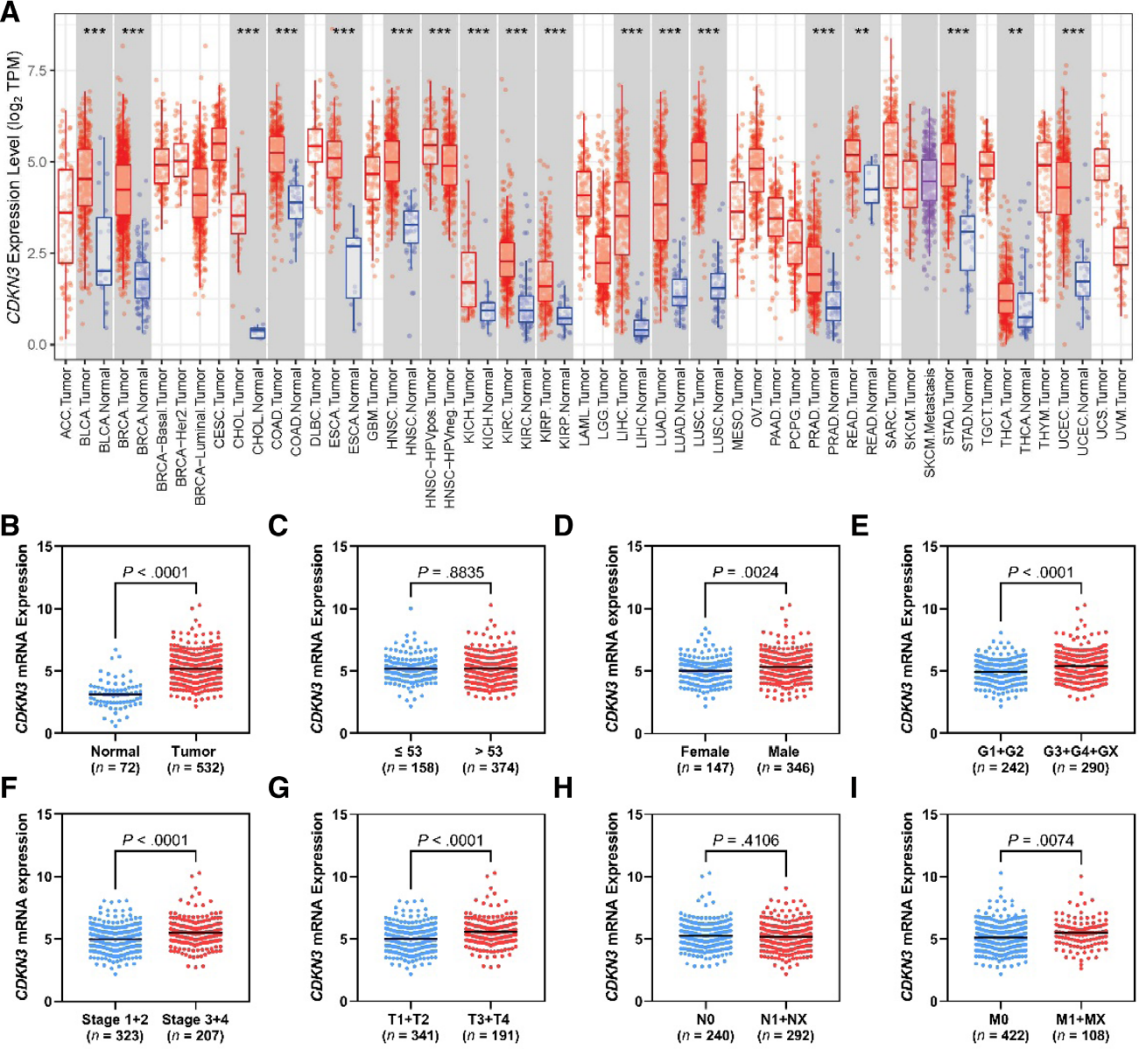
Pan cancer expression profile of *CDKN3* across TCGA cohorts in comparison to their associated NATs (A). Upregulated *CDKN3* expression in the TCGA ccRCC cohort compared to NAT (B). *CDKN3* expression correlation with dichotomized clinicopathological variables including age (C), gender (D), grade (E), stage (F), and TNM scoring (G–I). Data are presented as mean ± SEM. *P < .05, **P < .01, ***P < .001.

A pan-cancer analysis has been conducted to evaluate the expression of *CDKN3* in comparison to the corresponding normal tissues in the TCGA database (Fig. 1A). *CDKN3* has upregulated in bladder urothelial carcinoma, breast invasive carcinoma, cholangiocarcinoma, colorectal cancer, esophageal carcinoma, head and neck squamous cell carcinoma, liver hepatocellular carcinoma, lung adenocarcinoma, lung squamous cell carcinoma, prostate adenocarcinoma, rectum adenocarcinoma, stomach adenocarcinoma, thyroid cancer, and uterine corpus endometrial carcinoma. In addition, *CDKN3* expression followed the same trend in renal neoplasms including chromophobe (KICH), papillary, and clear cell renal cell carcinomas (KIRC). The latter is the focus of the current study.

### 3.2. *CDKN3* expression correlates with advanced ccRCC clinicopathological characteristics

*CDKN3* expression is significantly upregulated in ccRCC tissues compared to NAT (*P* < .0001, Fig. 1B). Its expression did not exhibit any remarkable difference across age groups (*P* = .8835, Fig. 1C), while male patients’ tissues possessed upregulated *CDKN3* compared to the female cohort (*P* = .0024, Fig. 1D). *CDKN3* expression was significantly linked to advanced disease features as in grade (grade 1 + 2 vs 3 + 4, *P* < .0001, Fig. 1E), stage (stage 1 + 2 vs 3 + 4, *P* < .0001, Fig. 1F), pT (T1 + T2 vs T3 + T4, *P* < .0001, Fig. 1G), and pM (M0 vs M1 + MX, *P* = .0074, Fig. 1I). On the contrary, lymph nodes involvement did not show evidence of *CDKN3* upregulation (N0 vs N1 + NX, *P* = .4106, Fig. 1H).

### 3.3. High *CDKN3* expression is associated with unfavorable prognosis in ccRCC cohort

**Figure 2.**
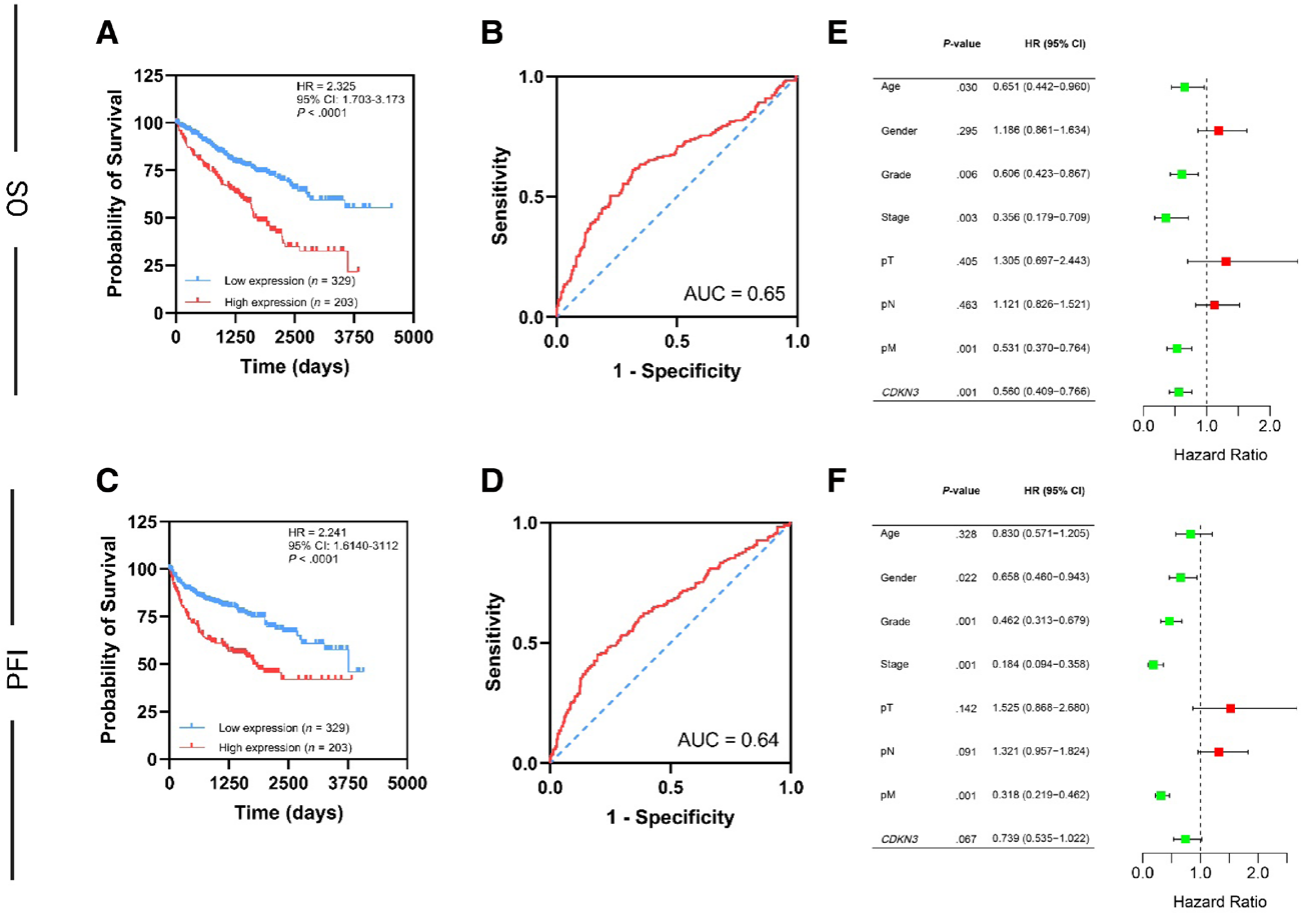
Kaplan–Meier survival curve illustrating that high *CDKN3* expression is correlated with shorten OS (A) in ccRCC cohort with acceptable predictability of the OS using ROC curves (B). Kaplan–Meier survival curve illustrating that high *CDKN3* expression is corelated with shorten PFI (C) in ccRCC cohort with acceptable predictability of the PFI using ROC curves (D). Forest plots presetting the multivariate Cox logistic regression analysis results in the OS (E) and PFI (F). ROC = receiver operating curve.

Kaplan–Meier analysis showed that high *CDKN3* expression is associated with shortened OS (HR: 2.325, 95% CI: 0.703–3.173, *P* < .0001) in comparison to the low expression group (Fig. 2A). In addition, *CDKN3* expression possesses a moderate predictability of the OS (AUC = 0.65) as shown in the receiver operating curve analysis (Fig. 2B). The same pattern has been observed in PFI, in which high *CDKN3* expression is associated with shortened PFI (HR: 2.241, 95% CI: 1.6140–3.112, *P* < .0001) (Fig. 2C) with similar predictability potential (AUC = 0.64) (Fig. 2D).

Univariate Cox logistic regression was utilized to evaluate the prognostic impact of *CDKN3* expression and the associated clinicopathological variables separately in the OS and PFI. The analysis showed that all the variables can predict the OS including the age (HR: 0.532, 95% CI: 0.367–0.771, *P* < .001), grade (HR: 0.388, 95% CI: 0.276–0.544, *P* < .001), stage (HR: 0.259, 95% CI: 0.189–0.356, *P* < .001), pT (HR: 0.316, 95% CI: 0.233–0.427, *P* < .001), pM (HR: 0.273, 95% CI: 0.201–0.371, *P* < .001), and *CDKN3* expression (HR: 0.426, 95% CI: 0.316–0.576, *P* < .001). Both gender (HR: 1.059, 95% CI: 0.779–1.441, *P* = .714) and pN (HR: 1.102, 95% CI: 0.819–1.482, *P* = .523) did not exhibit significant predictability. PFI univariate Cox logistic regression illustrated the same statistical trend in the following variables; age (HR: 0.667, 95% CI: 0.464–0.957, *P* = .028), gender (HR: 0.659, 95% CI: 0.465–0.932, *P* = .019), grade (HR: 0.285, 95% CI: 0.196–0.415, *P* < .001), stage (HR: 0.148, 95% CI: 0.104–0.212, *P* < .001), pT (HR: 0.223, 95% CI: 0.161–0.308, *P* < .001), pM (HR: 0.156, 95% CI: 0.113–0.215, *P* < .001), and *CDKN3* expression (HR: 0.443, 95% CI: 0.325–0.606, *P* < .001), except for the pN (HR: 1.138, 95% CI: 0.834–1.552, *P* = .415).

To test the absolute predictability of *CDKN3* expression in the OS and PFI with the elimination of confounding variables, multivariate Cox logistic regression was used (Fig. 2E and F). It showed that the following variables exhibit significant OS predictability, namely age (HR: 0.651, 95% CI: 0.442–0.960, *P* = .03), grade (HR: 0.606, 95% CI: 0.423–0.867, *P* = .006), stage (HR: 0.356, 95% CI: 0.179–0.709, *P* = .003), pM (HR: 0.531, 95% CI: 0.370–0.764, *P* = .001), and *CDKN3* expression (HR: 0.560, 95% CI: 0.409–0.766, *P* < .001). In contrast, gender (HR: 1.186, 95% CI: 0.861–1.634, *P* = .295), pT (HR: 1.305, 95% CI: 0.697–2.443, *P* = .405), and pN (HR: 1.121, 95% CI: 0.826–1.521, *P* = .463) predictability did not reach statistical significance. Figure 2E represents the visual representation of the OS multivariate Cox logistic regression (forest plot). PFI multivariate Cox logistic regression indicated that gender (HR: 0.658, 95% CI: 0.460–0.943, *P* = .022), grade (HR: 0.462, 95% CI: 0.313–0.679, *P* < .001), stage (HR: 0.182, 95% CI: 0.094–0.358, *P* < .001), and pM (HR: 0.318, 95% CI: 0.219–0.462, *P* < .001) were significantly different compared to age (HR: 0.830, 95% CI: 0.571–1.205, *P* = .328), pT (HR: 1.525, 95% CI: 0.868–2.680, *P* = .142), pN (HR: 1.321, 95% CI: 0.957–1.824, *P* = .091), and *CDKN3* expression (HR: 0.739, 95% CI: 0.535–1.022, *P* = .067) which did not exhibit any significance.

### 3.4. GSEA of high *CDKN3* expression group reveals multiple enriched pathways involved in its poor prognostic signature

**Figure 3.**
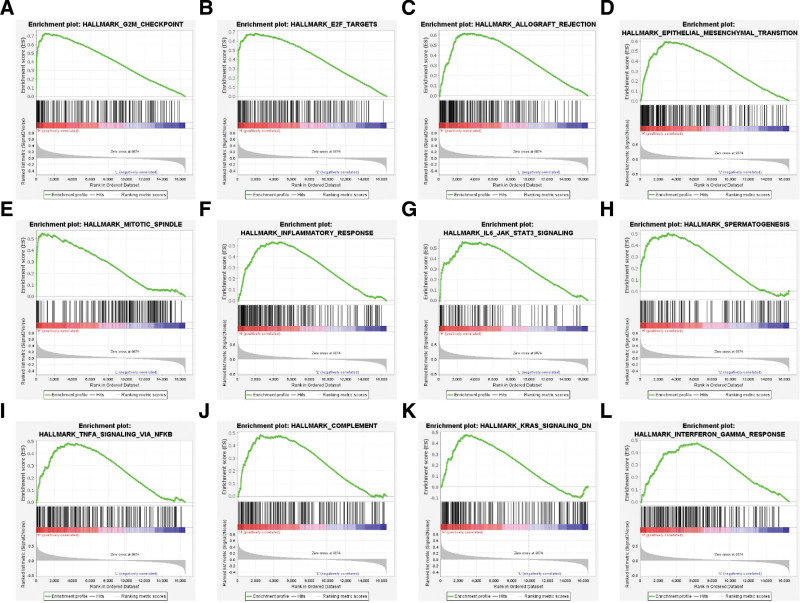
GSEA showing multiple enriched pathways within the C1 hallmarks of cancer dataset including G2M checkpoint (A), E2F targets (B), allograph rejection (C), epithelial-mesenchymal transition (D), mitotic spindle (E), inflammatory response (F), IL-6/JAK/STAT3 signaling (G), spermatogenesis (H), TNF-α signaling via NF-kB pathway (I), complement activation (J), KRAS signaling, coagulation (K), INF-γ signaling response (L). GSEA: gene set enrichment analysis.

GSEA was used to identify enriched pathways within the high *CDKN3* expression group in comparison to the low expression cohort using several gene sets (Fig. 3). C1 hallmarks of cancer gene illustrated an enrichment in 12 pathways including G2M checkpoint (ES = 0.730, Q < .001, FDR = 0, n = 187), E2F targets (ES = 0.683, Q < .001, FDR = 0, n = 192), allograph rejection (ES = 0.617, Q < .001, FDR = 0, n = 195), epithelial–mesenchymal transition (ES = 0.594, Q < .001, FDR = 0, n = 196), mitotic spindle (ES = 0.548, Q < .001, FDR = 0.0002, n = 195), inflammatory response (ES = 0.535, Q < .001, FDR = 0.0003, n = 198), IL-6/JAK/STAT3 signaling (ES = 0.562, Q < .001, FDR = 0.001, n = 87), spermatogenesis (ES = 0.505, Q < .001, FDR = 0.004, n = 131), TNF-α signaling via NF-kB pathway (ES = 0.484, Q < .001, FDR = 0.006, n = 198), complement activation (ES = 0.485, Q < .001, FDR = 0.006, n = 197), KRAS signaling (ES = 0.478, Q < .001, FDR = 0.006, n = 190), and INF-γ signaling (ES = 0.476, Q < .001, FDR = 0.007, n = 197).

### 3.5. *CDKN3*-related PPI map

**Figure 4.**
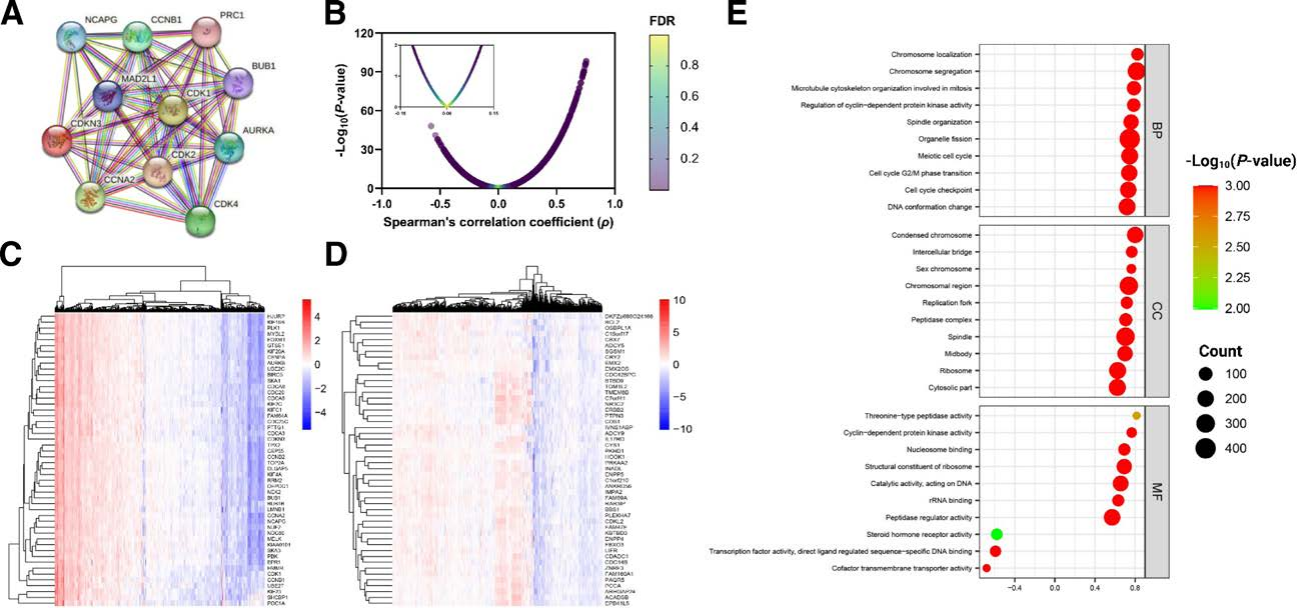
*CDKN3*-related PPI (A). *CDKN3*-related co-expressed genes (B) and their visual representations using heatmaps with similarity clustering (C and D). Functional annotations of *CDKN3*-related co-expressed genes using GO terms (E).

String was used to construct a *CDKN3*-related PPI map (Fig. 4A), which consists of 11 nodes with a combined score calculated the nodes and their corresponding combined score including CDK4 (0.508), CDK2 (0.616), CDKN3 (0.950), NCAPG (0.953), PRC1 (0.968), CDK1 (0.982), MAD2L1 (0.983), CCNA2 (0.987), CCNB1 (0.988), BUB1 (0.993), and NCAPG (0.988).

### 3.6. Biological functional clustering of *CDKN3* co-expressed genes revealed multiple pathways involved in its poor prognostic impact

LinkedOmics was utilized to identify positively and negatively significant *CDKN3* co-expressed genes within the TCGA ccRCC cohort (Fig. 4B–D) followed by functional annotation using GO (Fig. 4E) and KEGG terminology. The top 10 BPs enriched in the GO are chromosome segregation, microtubule cytoskeleton organization involved in mitosis, organelle fission, chromosome localization, spindle organization, meiotic cell cycle, cell cycle G2/M phase transition, regulation of cyclin-dependent protein kinase activity, cell cycle checkpoint, and protein localization to chromosome. In addition, the top 10 enriched CCs are condensed chromosome, chromosomal region, spindle, intercellular bridge, midbody, peptidase complex, replication fork, cytosolic part, ribosome, and Sm-like protein family complex. All the top 10 BPs and CCs were significantly enriched (*P* ≤ .05) with an FDR ≤ 0.05. In contrast, only 5 molecular functions achieved the significant threshold including structural constituent of ribosome, cyclin-dependent protein kinase activity, catalytic activity acting on DNA, nucleosome binding, and threonine-type peptidase activity. Co-expressed genes were also mapped to the following KEGG pathways involved in tumorigenesis of ccRCC including cell cycle, ribosome, p53 signaling pathway, proteasome, pyrimidine metabolism, cellular senescence, homologous recombination, DNA replication, drug metabolism, base excision repair, mismatch repair, and cytosolic DNA-sensing pathway. Through miRNA target prediction, across the significantly enrich targets non scored an FDR ≤ 0.05 except for a negatively enriched ACACTGG, MIR-199A, and MIR-199B.

### 3.7. *CDKN3* impacts tumor immune microenvironment and affects the expression of immune-related genes

**Figure 5.**
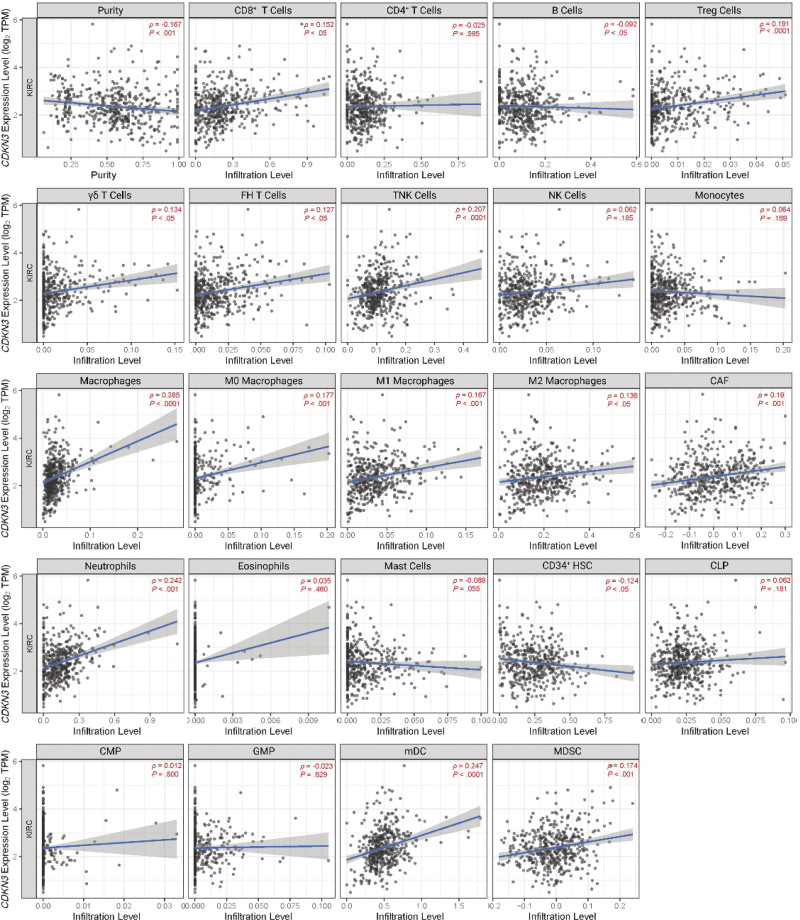
Immune cells infiltration in correlation to *CDKN3* expression using Spearman correlation coefficient (*ρ*). *P* ≤ .05 was considered statistically. Treg = regulatory T cells, FH = follicular helper, NKT = natural killer T cells, NK = natural killer, CAF = cancer associated fibroblasts, HSC = hematopoietic stem cell, CLP = common lymphoid progenitor, CMP = common myeloid progenitor, CMP = granulocyte-monocyte progenitors, mDC = myeloid dendritic cells, MDSC = myeloid-derived suppressor cells.

To investigate the tumor immune infiltration of ccRCC; TIMER server was utilized (Fig. 5) and a significant correlation between *CDKN3* expression and CD8^+^ cells (*ρ* = .154, *P* < .05), FH Tcells (*ρ* = .127, *P* < .05), γδ T cells (*ρ* = .134, *P* < .05), Tregs (*ρ* = .191, *P* < .0001), TNK cells (*ρ* = .207, *P* < .0001), macrophages (*ρ* = .177, *P* < .0001), M0 macrophages (*ρ* = .177, *P* < .001), M1 macrophages (*ρ* = .167, *P* < .001), M2 macrophages (*ρ* = .136, *P* < .05), CAF (*ρ =* .19, *P* < .001), neutrophils (*ρ* = .242, *P* < .001), mDCs (*ρ* = .247, *P* < .0001), CD34^+^ cells (*ρ* = -0.124, *P* < .05), and MDSC (*ρ* = .174, *P* < .001). In contrast nonsignificant correlations were observed in CD4^+^ T cells (ρ = −.025, *P* = .595), NK cells (ρ = .062, *P* = .185), monocytes (ρ = .064, *P* = .169), eosinophils (ρ = .035, *P* = .460), mast cells (ρ = −.089, *P* = .055), CLP cells (ρ = .062, *P* = .181), CMP (ρ = .012, *P* = .800), and GMP (ρ = −.023, *P* = .629).

Furthermore, a comprehensive analysis of the interrelationships between *CDKN3* expression and immune genes of various immune cell types, including but not limited to CD8^+^ T cells, T cells (general), B cells, monocytes, TAMs, M1 and M2 macrophages, neutrophils, NK cells and mDCs, in KIRC normal and KIRC NATs. For comparative analysis, we utilized kidney cortex tissue as the control (Table 1). The following genes were significantly correlated with *CDKN3*: *CCR7 (ρ* = 0.24, *P* < .0001), *CD2 (ρ* = 0.27, *P* < .0001), *CD3D (ρ* = 0.26, *P* < .0001), *CD3E (ρ* = 0.24, *P* < .0001), *CD8A (ρ* = 0.24, *P* < .0001), *CD8B (ρ* = 0.19, *P* < .0001), *CD19 (ρ* = 0.26, *P* < .0001), *CD68* (ρ = 0.21, *P* < .0001), *CD79A (ρ* = 0.18, *P* < .0001), *CD86 (ρ* = 0.27, *P* < .0001), *CD163 (ρ* = 0.32, *P* < .0001), *CSF1R (ρ* = 0.2, *P* < .0001), *IL10 (ρ* = 0.3, *P* < .0001), *IRF5 (ρ* = 0.095, *P* < .05), *ITGAM (ρ* = 0.14, *P* < .001), *KIR2DL4 (ρ* = 0.14, *P* < .001), *MS4A4A (ρ* = 0.27, *P* < .0001), *PTGS2 (ρ* = 0.15, *P* < .0001). *VSIG4 (ρ* = 0.26, *P* < .0001), *STAT4 (ρ* = 0.27, *P* < .0001), *STAT1 (ρ* = 0.3, *P* < .0001), *IFNG (ρ* = 0.27, *P* < .0001), *FOXp3 (ρ* = 0.3, *P* < .0001), *CCR8 (ρ* = 0.25, *P* < .0001), *TGFB1 (ρ* = 0.29, *P* < .0001) and *GZMB (ρ* = 0.21, *P* < .0001) and *CTLA4 (ρ* = 0.22, *P* < .0001) Additionally, *TBX21 (ρ* = 0.12, *P <* .05), *TNF (ρ* = 0.12, *P* < .05), *STAT6 (ρ* = -0.14, *P* < .05), *STAT5A (ρ* = 0.09, *P* value < .05), and *STAT5B (ρ* = 0.15, *P* < .05) show a relatively weak correlation with disease susceptibility. Moreover, the gene markers indicative of Th2 cells, M2 macrophages, Treg cells, and T cell exhaustion had the highest correlation with *CDKN3* were *GATA3 (ρ* = 0.34, *P* < .05), *CD163 (ρ* = 0.32, *P* < .0001), *TGFB1* (ρ = 0.29, *P* < .0001), and *LAG3* (ρ = 0.28, *P* < .0001), respectively.

**Table 1:** Correlation between *CDKN3* and immune-related genes in the TCGA ccRCC cohort, TCGA ccRCC NATS, and GTEx normal kidney cortex.

Immune cells	Gene markers	TCGA ccRCC		TCGA ccRCC NATs		GTEx kidney cortex	
		ρ	*P*-value	ρ	*P*-value	ρ	*P*-value
CD8 + T cell	CD8A	0.24	****	0.5	****	0.58	**
	CD8B	0.19	****	0.48	****	0.59	***
T-cell (general)	CD3D	0.26	****	0.58	****	0.54	**
	CD3E	0.24	****	0.51	****	0.59	**
	CD2	0.27	****	0.49	****	0.59	**
B-cell	CD19	0.26	****	0.43	***	0.48	*
	CD79A	0.18	****	0.39	***	0.61	***
Monocytes	CD86	0.27	****	0.63	****	0.63	****
	CSF1R	0.2	****	0.52	****	0.61	***
TAM	CCL2	0.026	.56	0.46	****	0.62	***
	CD68	0.21	****	0.42	***	0.63	***
	IL10	0.3	****	0.39	***	0.65	***
M1 macrophages	NOS2	0.037	.4	0.061	.61	0.33	.091
	IRF5	0.095	*	0.2	.087	0.41	*
	PTGS2	0.15	***	0.21	.077	0.1	.61
M2 macrophages	CD163	0.32	****	0.57	****	0.7	****
	VSIG4	0.29	****	0.62	****	0.69	****
	MS4A4A	0.27	****	0.57	****	0.57	**
Neutrophils	CEACAM8	−0.011	.8	0.1	.39	0.32	.092
	ITGAM	0.14	**	0.45	****	0.78	****
	CCR7	0.24	****	0.49	****	0.62	***
NK cells	KIR2DL1	0.02	.66	−0.023	.85	0.12	.53
	KIR2DL3	0.025	.57	0.23	.056	0.0056	.98
	KIR2DL4	0.14	**	0.13	.27	0.22	.27
	KIR3DL1	−0.079	.071	0.16	.17	0.11	.58
	KIR3DL2	−0.082	.062	0.17	.15	0.068	.73
	KIR3DL3	0.014	.75	0.08	.51	NA	NA
	KIR2DS4	−0.061	.17	0.13	.28	0.08	.69
DCs	HLA-DPB1	−0.049	.27	0.36	**	0.091	.64
	HLA-DQB1	−0.028	.34	0.34	**	−0.038	.85
	HLA-DRA	−0.033	.45	0.41	***	0.13	.49
	HLA-DPA1	0.045	.3	0.34	**	0.059	.77
	CD1C	−0.047	.29	0.37	**	0.053	.79
	NRP1	−0.06	.17	0.58	****	0.32	.094
	ITGAX	0.033	.45	0.63	****	0.18	.35
Th1	TBX21	0.12	**	0.5	.99	0.52	**
	STAT4	0.27	****	0.47	****	0.49	**
	STAT1	0.3	****	0.27	****	0.67	****
	INFG	0.27	****	0.22	*	−0.099	.62
	TNF	0.12	**	0.35	**	0.59	***
Th2	GATA3	0.34	**	0.15	.2	0.11	.58
	STAT6	-0.14	**	0.46	****	0.66	***
	STAT5A	0.09	*	0.04	**	0.69	****
	IL13	−0.002	.96	0.28	*	0.13	.51
FH T cells	BCL6	0.049	.26	0.33	**	0.61	***
	IL21	0.16	***	0.3	**	NA	NA
Th17	STAT3	0.071	.11	0.29	*	0.29	*
	IL17a	0.053	.22	0.28	*	0.11	.56
Treg cells	FOXP3	0.3	****	0.36	**	0.47	*
	CCR8	0.25	****	0.41	***	0.27	.17
	STAT5B	0.15	***	0.14	.24	0.59	***
	TGFB1	0.29	****	0.55	****	0.58	**
T cell exhaustion	PDCD1	0.21	****	0.26	*	0.66	***
	CTLA4	0.22	****	0.4	***	0.4	*
	LAG3	0.28	****	0.46	****	0.63	***
	HAVCR2	0.024	.58	0.15	.22	0.43	*
	GZMB	0.21	****	0.47	****	0.19	.34

DCs = dendritic cells, FH T cells = follicular helper T cells, NA = not applicable, NK cells = natural killer cells, TAM = tumor associated macrophages, Treg = regulatory T-cells.

* *P* < .05.

** *P* < .01.

*** *P* < .001.

*** *P < .0001.

The following correlations didn’t reach statistical significance including *CCL2 (ρ* = .026, *P* = .56), *CD1C (ρ* = −.047, *P* = .29), *CEACAM8 (ρ* = −.011, *P* = .80), *HLA-DPA1 (ρ* = −.049, *P* = .27), *HLA-DPB1 (ρ* = −.028, *P* = .34), *HLA-DRA (ρ* = −.033, *P* = .45), *HLA-DQB1 (ρ* = −.028, *P* = .34), *ITGAX (ρ* = .033, *P* = .45) *NOS2 (ρ* = .037, *P* = .40), *IL13 (ρ* = −.002, *P* = .96), *BCL6 (ρ* = .049, *P* = .26), *STAT3 (ρ* = .071, *P* = .11) and *IL17a* (ρ = .053, *P* = .22), *HAVCR2 (ρ* = .024, *P* = .58), *NRP1 (ρ* = −.06, *P* = .17), *KIR2DL1 (ρ* = .02, *P* = .66), *KIR2DL3 (ρ* = .025, *P* = .57), *KIR2DS4 (ρ* = −.061, *P* = .17), *KIR3DL1 (ρ* = −.079, *P* = .07), *KIR3DL2 (ρ* = −.082, *P* = .06), and *KIR3DL3 (ρ* = 0.014, *P* = .75).

## 4. Discussion

In the current work, *CDKN3* has been portrayed as a prognostic biomarker in ccRCC through a comprehensive bioinformatics analysis using the TCGA database. *CDKN3* is associated with shortened OS and poor prognosis in ccRCC. Enrichment analysis has highlighted the following pathways to be affected by *CDKN3* upregulation, including allograph rejection, epithelial–mesenchymal transition, mitotic spindle, inflammatory response, IL-6/JAK/STAT3 signaling, spermatogenesis, TNF-α signaling via NF-kB pathway, complement activation, KRAS signaling, and INF-γ signaling. In addition, *CDKN3* expression correlated significantly with many immune cellular components and immune-related genes. Although the main role of *CDKN3* is to inhibit the progression of the cell cycle, it is overexpressed in many types of malignancies such as gastric cancer, prostate cancer, hepatocellular, cervical, breast, and epithelial ovarian cancers which was in concomitant with expression pan-cancer analysis conducted.^[18,39–42]^ Overexpression of *CDKN3* in these malignancies is associated with inadequate prognosis, while *CDKN3* in glioblastomas is considered a tumor suppressor gene. A study showed that CDKN3 prevents aneuploidy and it performs the complex task of managing cell division through a combination of direct pathways that involve regulating the SAC and the mitotic clock, as well as indirect mechanisms that involve preserving the integrity of centrosomes.^[28,43]^

A variety of studies found that CDKN3 played a crucial role in tumor progression. A study performed on esophageal carcinoma revealed that CDKN3 enhances tumor progression and chemoresistance against cisplatin through modulation and interaction of RAD51.^[23]^ Moreover, silencing or inhibiting of *CDKN3* reduces the migration of breast cancer cells.^[22]^ Furthermore, silencing also increases apoptosis in gastric cancer cell lines and reduces survival by lowering the expression of different cell-regulating proteins such as CDK2, CDC25, and CCNB1/2 thus inhibiting proliferation and invasion of gastric cancer cells.^[42]^ Moreover, *CDKN3* knockdown elicits an effect on human ovarian cancer cells, manifesting as a substantial reduction in their proliferative and invasive capabilities, concurrently with a remarkable elevation in apoptosis.^[44]^ In addition, *CDNK3* silencing in nasopharyngeal carcinoma subdues its ability to proliferate. Hence, affecting carcinogenesis through the modulation of p27. It also decreased the phosphorylation of AKT while increasing the activated form of caspase 3 hence, increasing apoptosis in nasopharyngeal carcinoma.^[45]^ A recent work done on esophageal squamous cell carcinoma confirmed that the mechanism involved in the increase of tumor characteristics by CDKN3 such as migration, invasion, and cell proliferation was modulated through the activation of the AKT pathway. When *CDKN3* was silenced, inhibited or knocked, it suppressed the migration and cellular proliferation in addition to invasion in esophageal squamous cell carcinoma.^[46]^ Downregulation in the expression levels of *CDKN3* was found in high-stage HCC that have immature tumor cells. Paradoxically, knocking down *CDKN3* increased the elements of AKT pathway with its downstream targets such as p21/p53 leading to cell survival. Furthermore, culture cells mimicking HCC with *CDKN3* knocked down tolerated cisplatin more than cells expressing it.^[47]^ Thus, confirming the variation in *CDKN3* expression in different types of malignancies. On the other hand, a more recent paper showed that there was a notable association between the expression of *CDKN3* in HCC and a substantial increase in the OS time. Moreover, *CDKN3* levels were increased compared to adjacent para-carcinoma tissues. Furthermore, upregulation of *CDKN3* expression is positively associated with an increased incidence of HCC-related mortality. Macro- and micro-invasion were analyzed to explore the relationship with the levels of CDKN3. HCC with vascular invasion showed higher levels of CDKN3. A positive relationship with CTNNB1 was also observed. The same article showed that suppressing CDKN3 promotes the proliferation of HCC cells and confers a diminished sensitivity to Adriamycin chemotherapy.^[48]^ Overexpression of *CDKN3* is associated with poor progression in prostate cancer and its inhibition significantly reduces cancer cell growth. It induced cells into G0/G1 arrest and apoptosis. Moreover, it inhibited the invasion of prostate cancer cells and lowered the expression of MCM2/MCM3, PCNA, and CDK2. Also, a mice model xenograft transfected with siRNA hindered the tumor progression of androgen-independent prostate cancer cells in an in vivo setting.^[42]^ All the aforementioned findings were observed through gene enrichment and co-expression analysis.

Although, the role of CDKN3 in RCC has been previously discussed through wide genetic associations and experimental results; its prognostic and mechanist role are not well understood. *CDKN3* overexpression xenograft model experiments found that tumors extracted from overexpressing *CDKN3* cells were greater in size than the mock experiment.^[25]^ ZNF677 is a zinc finger protein-coding gene. It has recently been shown that its upregulation downregulates and inhibits CDKN3 through interaction with its promoter. As reported in the same study, CDKN3 correlated with poor prognosis and survival in RCC, and when inhibited, reduced prefiltration rates and increased apoptosis were observed in RCC cell lines. Furthermore, RCC cell lines *CDKN3* knocked down had a reduced tumor size compared to the mock injection.^[49]^ Yin-Yang 1 (YY1) is a protein that is involved in transcriptional regulation and many biological processes such as proliferation, differentiation, and apoptosis. A study found a direct association between YY1 and CDKN3 in pancreatic cancer. Additionally, YY1 inhibited proliferation and migration by downregulating CDKN3 and higher CDKN3 levels correlated negatively with survival and prognosis. Additionally, overexpression of YY1 inhibited the p21/p53 pathway via CDKN3.^[50]^ CDKN3 is increased in BLC and correlated with poor prognosis and survival compared to BLC patients with low expression of CDKN3. Moreover, inhibition of CDKN3 showed lower cell proliferation, migration, and arrested cells in M0/M1 phases. CRC tissues had higher levels of CDKN3 expression than tissues surrounding CRC. It contributed to cellular proliferation and migration, as proven when *CDKN3* was knocked down it had a lower colony-forming capacity and proliferation rate. Furthermore, *CDKN3* expression had a negative correlation with tumor necrosis factor-α-induced protein 8-like 1. CDKN3 was found to modulate the resistance of CRC cells to cisplatin, thereby driving their proliferation. This was accomplished through the regulation of tumor necrosis factor-α-induced protein 8-like 1 by CDKN3.^[40]^ Another finding about CDKN3 involvement in cisplatin resistance in BLC showed that CDKN3 altered/inhibited GC chemoresistance in BLC cell lines.^[39]^

GSEA identified a negatively enriched ACACTGG, MIR-199A, and MIR-199B to be affected by *CDKN3* upregulation. The relationship between CDKN3 and miRNA is very well understood. miR-181a-5p is of a particular importance because in recent years it was found that it suppresses non-small cell lung carcinoma. There is a theoretical possibility that hsa-miR-181d-5p may regulate *CDKN3* expression through the AKT signaling pathway, thereby potentially influencing the progression of non-small cell lung carcinoma. Additionally, CDKN3 was found to be expressed at high levels, in contrast to has-miR-181d-5p, which showed low levels of expression. Sequence prediction software showed has-miR-181d-5p has a binding site and interacts with the CDKN3 3′UTR. Furthermore, upregulation of has-miR-181d-5p or silencing/inhibiting CDKN3 demonstrated inhibition of cellular proliferation, increased apoptosis, inhibition of cellular migration/invasion, repression of the Akt signaling pathway, and inhibiting tumor growth in mice.^[51]^

The study has multiple limitations mainly presented by its retrospective nature and moderate sample size. In addition, the TCGA database present a bulk RNA sequence data lacking the single cell RNA sequencing input for more precise analysis. The detailed molecular mechanisms behind the *CDKN3* poor prognostic signature requires rigorous experimental evidence. Based on the available data, the impact of *CDKN3* upregulation in modulating tumor immune microenvironment is still vague and requires further exploration.

## 5. Conclusion

As an important element of cell cycle regulatory proteins; *CDKN3* has been identified as a poor prognostic biomarker in ccRCC through bioinformatics analysis utilizing the TCGA cohort. GSEA highlighted several impacted molecular pathways. Immune correlations suggest that *CDKN3* influences tumor immune microenvironment and modulates immune-genes expression.

## Acknowledgments

The leading author would thank the 6th year medical school surgery final exam for giving the time and inspiration to learn the basis of bioinformatics which was the core nucleus to this work.

## Author contributions

**Conceptualization:** Ahmed Hesham Al Sharie, Tamam El-Elimat, Yazan O. Al Zu'bi, Feras Q. Alali.

**Data curation:** Abdulmalek M. Abu Zahra, Balqis M. Abu Mousa, Mohammad S. Bani Amer, Kinda Al-Kammash, Alma Abu Lil, Abubaker A. Al Malkawi, Zainab Alazzeh.

**Formal analysis:** Ahmed Hesham Al Sharie, Reem F. Darweesh, Ayah K. Al-Khaldi, Balqis M. Abu Mousa, Mohammad S. Bani Amer, Kinda Al-Kammash, Yazan O. Al Zu'bi, Alma Abu Lil, Abubaker A. Al Malkawi.

**Funding acquisition:** Feras Q. Alali.

**Investigation:** Tamam El-Elimat, Mohammad S. Bani Amer, Alma Abu Lil.

**Methodology:** Ahmed Hesham Al Sharie, Abdulmalek M. Abu Zahra, Reem F. Darweesh, Ayah K. Al-Khaldi, Balqis M. Abu Mousa.

**Project administration:** Tamam El-Elimat, Feras Q. Alali.

**Resources:** Tamam El-Elimat.

**Software:** Balqis M. Abu Mousa, Mohammad S. Bani Amer, Kinda Al-Kammash.

**Supervision:** Ahmed Hesham Al Sharie, Tamam El-Elimat, Balqis M. Abu Mousa, Feras Q. Alali.

**Validation:** Abdulmalek M. Abu Zahra, Reem F. Darweesh, Ayah K. Al-Khaldi, Yazan O. Al Zu'bi, Abubaker A. Al Malkawi.

**Visualization:** Abdulmalek M. Abu Zahra, Kinda Al-Kammash, Alma Abu Lil.

**Writing – original draft:** Ahmed Hesham Al Sharie, Abdulmalek M. Abu Zahra, Tamam El-Elimat, Reem F. Darweesh, Ayah K. Al-Khaldi, Zainab Alazzeh.

**Writing – review & editing:** Tamam El-Elimat, Feras Q. Alali.
